# A Review of Lumbar Radiculopathy, Diagnosis, and Treatment

**DOI:** 10.7759/cureus.5934

**Published:** 2019-10-17

**Authors:** James A Berry, Christopher Elia, Harneel S Saini, Dan E Miulli

**Affiliations:** 1 Neurosurgery, Riverside University Health System Medical Center, Moreno Valley, USA; 2 Neurology, Alleghany Health Network, Pittsburgh, USA

**Keywords:** lumbar radiculopathy, spine neurosurgery, lumbar spine

## Abstract

We review the epidemiology, etiology, symptomatology, clinical presentation, anatomy, pathophysiology, workup, diagnosis, non-surgical and surgical management, postoperative care, outcomes, long-term management, and morbidity of lumbar radiculopathy. We review when outpatient conservative management is appropriate and "red flag" warning symptoms that would necessitate an emergency evaluation. Diagnostic modalities, including magnetic resonance imaging (MRI), computerized tomography (CT), contrast myelogram, electromyogram (EMG), and nerve conduction velocity (NCV), are involved in the diagnosis and decision-making are discussed. Treatment of lumbar radiculopathy requires a multimodal and multispecialty team. We review indications for the involvement of other professionals, including physical therapy (PT), occupational therapy (OT), physical and rehabilitation medicine (PMR), and pain management.

## Introduction and background

Lumbar radiculopathy is one of the most common complaints evaluated by a spine surgeon. Its prevalence has been estimated to be 3%-5% of the population, affecting both men and women. Age is a primary risk factor, as it occurs secondary to the degenerative process within the spinal column. Symptoms typically begin in midlife, with men often affected in the 40s while women are affected in the 50s and 60s [1-2]. Females have a higher risk in certain populations, with physically demanding careers such as service in the military. In the general population, there is a male preponderance [3]. Degenerative spondyloarthropathies are the primary cause of lumbar radiculopathy [1]. Patients commonly present with back pain that is associated with their radiculopathy. By definition, radiculopathy describes pain that radiates down the legs and is often described by patients as electric, burning, or sharp. The most common underlying cause of radiculopathy is irritation of a particular nerve, which can occur at any point along the nerve itself and is most often a result of a compressive force. In the case of lumbar radiculopathy, this compressive force may occur within the thecal sac, as the nerve root exits the thecal sac within the lateral recess, as the nerve root traverses the neural foramina or even after the nerve root as exited the foramina. It may be related to disc bulging or herniation, facet or ligamentous hypertrophy, spondylolisthesis, or even neoplastic and infectious processes. The diagnosis of the causative agent and subsequent treatment starts with a thorough physical exam.

## Review

Diagnosis

**Figure 1:**
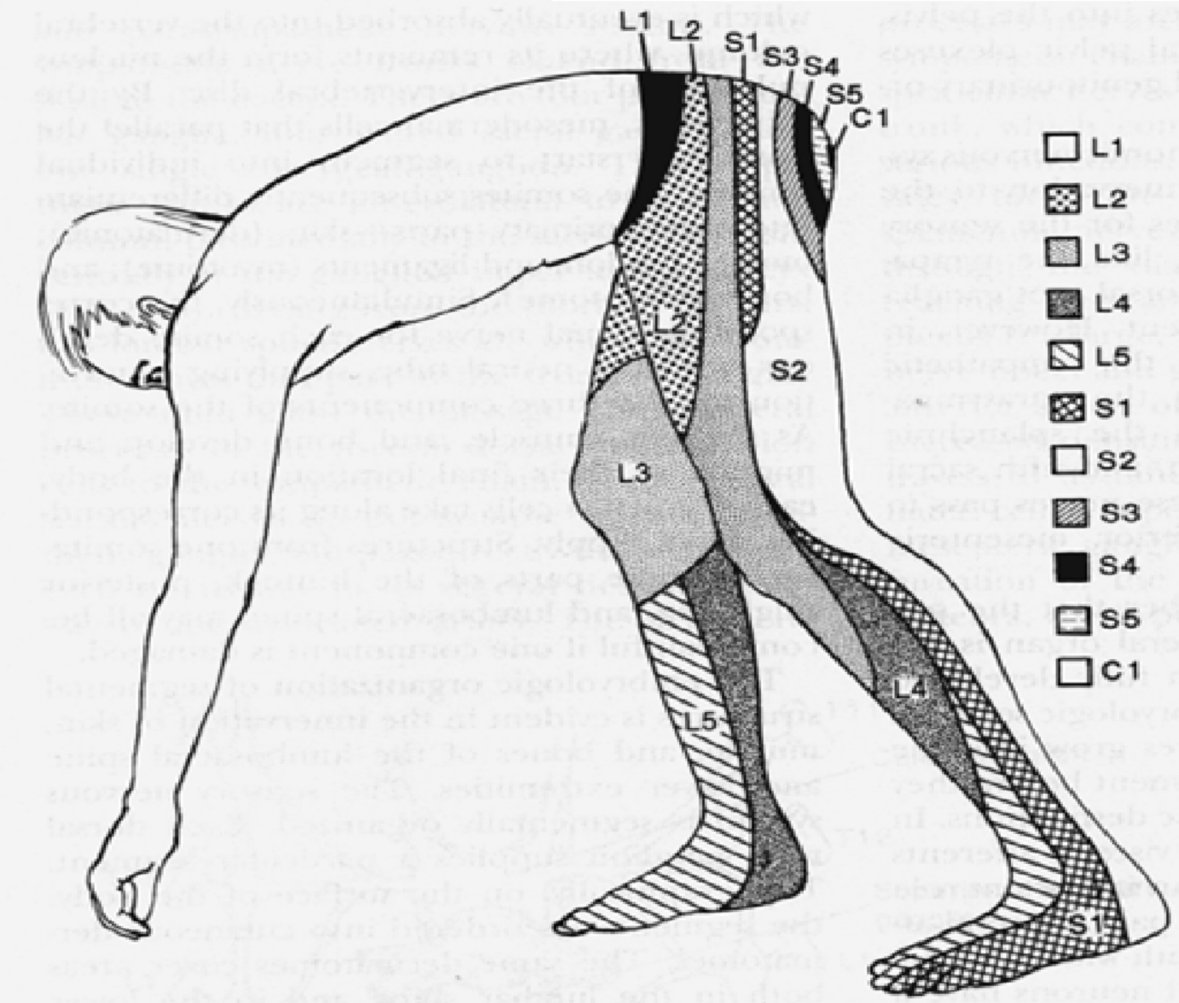
Dermatomes Anatomical map of the sensory dermatomes of the Lumbosacraloccygeal region Image provided by the National University of Córdoba with permission for use.

The initial exam should include a complete history and physical exam, including manual muscle testing, sensory testing, deep tendon reflexes, and Lasegue’s sign [4]. Lasegue’s sign is assessed with the patient lying in the supine position, the knee extended, the ankle dorsiflexed, and the cervical spine flexed. The examiner lifts the patient’s lower extremity off the table towards 90 degrees, which will elicit radicular pain as the nerve root is stretched. Classically, when radiculopathy is caused by nerve root compression pain, sensory loss occurs in a dermatomal pattern (Figure 1). Motor loss may occur in a myotomal pattern (Table 1). The distribution of pain and motor findings on physical exam should guide the neurosurgeon to the region of the spine to focus on, with further modalities such as MRI and electrodiagnostic testing.

**Table 1:** Lumbosacral myotomes Anatomical distribution of lumbosacral myotomes

Spinal Nerve	Myotome
L2	Hip Flexion Iliopsoas
L3	Knee Extension
L4	Ankle Dorsiflexion Tibialis Anterior
L5	Ankle Eversion (peronous longus and brevis) Great Toe Extension Extensor Hallucis Longus
S1	Plantar Flexion Gastrocnemius, Soleus

**Figure 2:**
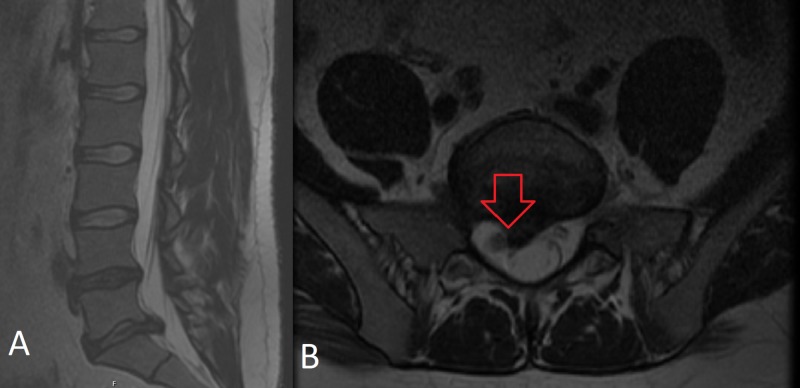
A. T2 sagittal MRI of the lumbar spine w/o contrast B. Axial MRI of the lumbar spine w/o contrast A. Sagittal T2 w/o contrast MRI lumbar spine shows a large 9 mm L5/S1 paracentral disc protrusion with mass effect on the thecal sac. B. Axial T2 w/o contrast MRI lumbar spine; the same patient shows compression of the right exiting S1 nerve root, which has caused this patient to experience right S1 radiculopathy.

After a thorough physical exam, diagnostic imaging should be reviewed. The optimal imaging modality for the evaluation of radiculopathy is MRI of the lumbar spine without contrast, which can show compression of the nerve root (see Figure 2). Contrast-enhanced MRI may be useful or indicated in cases where a tumor, infection, or prior surgery has occurred. In cases where MRI is not available or possible, a CT myelogram is a reasonable alternative.

Clinical Pearl

Ensure that you evaluate the MRI with the patient’s clinical exam in mind. Often, a far lateral disc herniation can be missed when not specifically looking for it. MRI is a triplanar modality that necessitates utilizing the axial, sagittal, and coronal sequences. The sagittal sequences can demonstrate far lateral disc herniations with the foramina. The coronal sequence shows nerve roots and foraminal and extraforaminal regions where a far lateral disc herniation occurs.

When there is a lack of correlation between the exam findings and imaging studies, electrodiagnostic testing may be employed. Electromyography (EMG) and nerve conduction velocities (NCV), as well as somatosensory evoked potentials (SSEP), can help differentiate between radiculopathy and more diffuse disorders of the peripheral nervous system. It is important to note that while EMG and NCV studies may be a useful diagnostic tool when combined with a thorough history, clinical examination, and other diagnostic studies, they do have limitations and potential pitfalls. EMG and NCV studies are affected by the patients’ level of cooperation, which may be limited by pain, temperature of room, electrolyte and fluid balance, pre-existing medical comorbidities, such as diabetes mellitus, thyroid disease, or renal failure that can produce peripheral neuropathy, medications such as statins, which can produce myopathy, movement disorders that produce tremors, prior surgeries, such as laminectomy, which may give paraspinous muscle false-positives, body habitus with extreme obesity preventing the full insertion of needles into muscle, congenital anatomical variations, for example, Martin-Gruber nerve anastomosis, and subjective interpretation of the data by the individual clinician [5]. Diagnostic nerve root blocks may also help to localize the symptomatic level [6].

Treatment

Non-Surgical

The need for surgical intervention, the timing of surgery, and surgical approaches has been extensively studied, yet, controversy still exists. Guidelines for approaching lumbar radiculopathy favor an initial trial of conservative management, including patient education, staying active/exercise, manual therapy (such as McKenzie exercises), and non-steroidal anti-inflammatory drugs (NSAIDs) as first-line treatments [7-9]. The use of McKenzie exercises has been demonstrated to provide some acute symptomatic relief in patients undergoing conservative management for lumbar radiculopathy [10]. Oral corticosteroids prescribed as a taper may benefit patients in the acute phase [11]. Often, the next step in treatment is pain injections, which may include epidural steroid injections, facet injection, or transforaminal injections, which have been shown to provide long-term relief of symptoms [12]. These injections typically consist of a combination of an anti-inflammatory agent, such as a glucocorticoid, and a long-lasting anesthetic such as Marcaine. In situations where the pain generator is indeterminate, spinal injections can be both diagnostic and therapeutic. For example, a patient with significantly low back pain and some numbness in her left foot who has extensive arthritic changes throughout her spinal column receives an injection in her facets with profound symptomatic relief. The injection into her lumbar spinal facet joints provided her with significant symptomatic relief, demonstrating that arthritic changes in her facet joints, not lumbar radiculopathy from a compressed nerve root, were her pain generator. However, a patient with significant lower extremity pain in an L5 dermatomal pattern experiences significant relief after an epidural injection, indicating the pain generator is likely the compressed left L5 nerve root, not arthritic changes in the facet joints.

Surgical Decision-Making

When conservative treatments fail to provide symptom relief, surgical intervention is considered. The timing between surgery and when conservative measures can be designated as failed therapy typically ranges between four and eight weeks [13]. However, the question as to who would benefit from surgery and who should continue conservative therapy is debatable. The Spine Patient Outcomes Research Trial (SPORT) trail attempted to answer this question [14]. It evaluated 501 patients with herniated lumbar discs and compared surgical vs. non-surgical treatments. The primary outcome being SF-36, which is a benefit-cost ratio of lumbar fusion in comparison to other surgical Interventions and Oswestry Disability Index (ODI) scores at specific intervals. In the end, it found that both the surgery and the non-operative treatment groups improved substantially over a two-year period, with improvements consistently in favor of surgery for all periods but that were small and not statistically significant [14].

A significant contribution from this study was that most patients improve given time, either with or without surgery. At eight years after symptom onset, those patients who benefited most from a surgical intervention were patients with sequestered disc fragments, symptom duration of greater than six months, those with higher levels of low back pain, or who were neither working nor disabled at baseline [4].

The ultimate timing of surgery is often based on the severity of the patient’s symptoms and clinical experience. Overall, surgery has been shown to be of benefit to patients with more severe symptoms [15].

Surgical Techniques

The gold standard surgical procedure for simple lumbar disc herniation remains a discectomy. In 1939, Semmes presented a subtotal laminectomy and retraction of the dural sac to remove the herniated disc [16]. Since then, many iterations of this procedure focussing on less invasive techniques have been developed. In 1977 and 1978, Caspar and Williams reported refinements in the approach with the use of a microsurgical technique [17]. In 1997, Foley introduced the microendoscopic discectomy (MED) procedure [18-19].

Surgical options include open laminectomy with discectomy, the so-called “mini-open” hemilaminectomy with a microdiscectomy, minimally invasive hemilaminectomy with microdiscectomy via tubular retractors, and MED. Studies have shown MED to be superior to open surgical techniques in producing less irritation of the nerve by intraoperative EMG studies [20], less requirement of postoperative analgesia during the hospital stay, less mean operative blood loss, and a lower mean number of rest days [21-22]. Less invasive methods may also produce less joint destabilization due to less destructive techniques as well as decreased surgical and hospital costs [22]. Minimally invasive techniques are not without limitations such as a restricted cone field of vision for the surgeon and inability to approach pathology from other angles. Minimally invasive techniques may be appropriate under the correct conditions and should be evaluated on a case-by-case basis.

For traditional open discectomy, localization of the level is first obtained with fluoroscopy and a midline incision is made at the level of the disc. The incision is then continued down in a subperiosteal fashion to expose the lamina of the upper level and ligamentum flavum over the interspace laterally. A retractor is then placed. The microscope is then brought in and a hemilaminectomy and partial medial facetectomy is performed with a high-speed drill and Kerrison Roungeurs. The ligamentum is then detached from the lamina and removed, exposing the nerve root crossing over the disc. The root and thecal sac are retracted medially and the annulus exposed. A box incision in the disc annulus is made and disc material removed. A nerve hook can be used to sweep anterior to the thecal sac to retrieve any herniated fragments. Loose fragments within the disc space can be flushed out from the disc space with irrigation. The advantages of this approach are increased visualization, the ability to use a wider variety of instruments, better visualization, and the ability to approach pathology from multiple trajectories not limited to a specific trajectory such as minimally invasive surgery (MIS) approaches.

For a minimally invasive (MIS) approach, a paramedian incision 2 centimeters off the midline is made. A small stab incision is made in the lumbodorsal fascia and a K-wire or initial tubular retractor is docked on the facet joint at the level of the disc space. A muscle splitting approach is utilized by the introduction of a series of sequential dilators. The microscopic is then brought in or in the case of MED, a rigid endoscope is inserted. The laminar facet junction is visualized and laminotomy, medial facetectomy, and microdiscectomy are performed in a similar fashion to the open technique.

Clinical Pearl

When using the MIS approach, it is essential to direct the trajectory of the tube perpendicular to the disc of interest. An altered trajectory will limit the ability to fully visualize and surgically decompress this nerve root. Confirm the trajectory with fluoroscopy.

## Conclusions

Lumbar radiculopathy is one of the most common neurological complaints to be evaluated by a neurosurgeon practicing in a rural environment. While the pathology has not changed, newer, less invasive techniques are being developed to surgically treat these patients in the evolving field of spine surgery. Intimate knowledge of the signs, symptoms, red-flag warning signs, radiographic imaging, diagnostic tools, and conservative and surgical interventions is a necessity. The red-flag warning signs that would prompt an emergent evaluation include saddle anesthesia, incontinence to bowel or bladder, and sudden paresis in an extremity.
